# Purified Clinoptilolite-Tuff as an Efficient Sorbent for Gluten Derived from Food

**DOI:** 10.3390/ijms23095143

**Published:** 2022-05-05

**Authors:** Carmen Ranftler, Andreas Röhrich, Andreas Sparer, Cornelius Tschegg, Dietmar Nagl

**Affiliations:** Glock Health, Science and Research GmbH, Hausfeldstraße 17, 2232 Deutsch-Wagram, Austria; carmen.ranftler@glock.at (C.R.); andreas.roehrich@glock.at (A.R.); andreas.sparer@gmail.com (A.S.); cornelius.tschegg@glock.at (C.T.)

**Keywords:** zeolite, celiac disease, prolamin, gluten, gliadin, wheat allergy, clinoptilolite, dietary supplement, ELISA, artificial fluids

## Abstract

Various gluten-related diseases (celiac disease, wheat allergy, gluten sensitivity) are known and their incidence is growing. Gluten is a specific type of plant storage protein that can impair the health of gluten-prone persons following consumption, depending on the origin. The most severe effects are induced by wheat, barley, and rye. The only treatment is based on the absolute avoidance of those foods, as even traces might have severe effects on human well-being. With the goal of binding gluten impurities after ingestion, an in vitro setting was created. A special processed kind of zeolite, purified clinoptilolite-tuff (PCT), was implemented as an adsorber of gluten derived from different origins. Zeolites are known for their excellent sorption capacities and their applications in humans and animals have been studied for a long time. Tests were also performed in artificial gastric and intestinal fluids, and the adsorption capacity was determined via a certified validated method (ELISA). Depending on the kind of gluten source, 80–130 µg/mg of gluten were bound onto PCT. Hence, purified clinoptilolite-tuff, which was successfully tested for wheat, barley, and rye, proved to be suitable for the adsorption of gluten originating from different kinds of crops. This result might form the basis for an expedient human study in the future.

## 1. Introduction

### 1.1. Gluten—Origin, Definition, and Possible Health Problems

In ancient times, members of the grass family (Poaceae) were cultivated, resulting in what we know today as the cereal crops [1]. They contain storage proteins—used for nourishing the growing plant embryo—in amounts of up to 8–15% of total flour weight [2]. Many of them further incorporate special storage proteins (i.e., a group of related prolamin proteins that account for up to 50–80% of the total grain protein in wheat, barley, and rye) that are problematic for people affected by different gluten-related diseases [2,3]. These diseases can be classified into three different pathologies [1]: celiac disease (a chronic autoimmune disorder of the small intestine triggered by gluten and gluten-related proteins, characterized by a specific genotype, as well as autoantibodies); allergy to wheat (i.e., an IgE-mediated immune response to various wheat proteins, not limited to gluten proteins); and non-celiac gluten sensitivity (which is not mediated by the IgE or autoimmune mechanisms, and which usually does not lead to typical intestinal damage). Celiac disease currently affects 0.5–1% of the world population, with an increasing tendency, while non-celiac gluten sensitivity was reported in about 6% of the population (0.16–13%, with high variability between studies). Additionally, wheat allergy is prevalent in 0.33–1.17% of the population, and is estimated to affect 10–25% of patients with food allergies [1,4]. 

Celiac disease affects the whole human body. Due to malnutrition induced by an immune reaction against a sequence of amino acids found in prolamins, the small intestine changes pathologically, resulting in deformation of the villi (which are responsible for absorption of nutrients) [1]. The term “prolamins” derives from the most common amino acids found in these proteins, which are rich in proline and glutamine (≤25% and ≤45% of the total amounts, respectively). The same applies for the name “glutelins”, which is a composition of glutamine and proline, two amino acids that are highly resistant to human gastrointestinal digestion and responsible for triggering an immune response when coming into contact with the mucosa of genetically predisposed persons [1,2]. There is no cure for these enteropathies; patients must strictly obey a gluten-free diet, which was introduced as standard therapy in the 1950s. Unfortunately, this special diet is low in vitamins and minerals and might lead to nutritional deficiencies over time. On the other hand, gluten intoxication causes serious health problems—gastrointestinal cancer, osteoporosis, mental disorders, and infertility are among the most common [1,4]. Hence, patients suffering from prolamin intolerance must not consume gluten-containing food [1,4].

Most often, prolamin proteins are simply called “gluten” (from glutenins in wheat). Depending on their species, their names (Table 1) vary. Accordingly, in wheat (*Triticum aestivum*), they are called gliadins (monomeric) and glutenins (polymeric); in barley (*Hordeum vulgare*), their name is hordeins; in rye (*Secale cereale*), they are called secalins; and in oats (*Avena sativa*), they are referred to as avenins. Other crops, such as rice (*Oryza sativa*, oryzins), corn (*Zea mays*, zeins), sorghum (*Sorghum bicolor*, kafirins), millet (*Eleusine coracana*), and teff (*Eragrostis tef*), also contain gluten proteins [1,4,5].

Prolamins can cause different degrees of severity of enteropathies, depending on the plant they are derived from. Thus, a gluten-free diet strictly avoids wheat, rye, and barley. In some patients, avenin can also inflict health problems, but most often such problems derive from oats which are contaminated with barley or wheat gluten [2]. Generally, under a gluten-free diet, the highest risk of coming into contact with cross-contaminated food is via consumption of products that were originally gluten free, but that have significant gluten impurities due to harvesting, production, processing, packing, and storing [6].

As a result of the severe effects of prolamins in food, a labeling requirement was initiated more than 40 years ago. In the European regulation, the Codex Alimentarius [7], <100 ppm is considered to be “very low gluten”, whereas 20 ppm and below corresponds to “gluten free” and such foods are considered safe for patients exhibiting celiac disease to consume [7,8]. The beginning of gluten-free food regulation dates back to 1979, with the first publication of the Codex Alimentarius. Since then, it has constantly been revised and amended [7,8].

### 1.2. Zeolite—Chemical Composition, Use and Purification

Zeolites are minerals belonging to the group of alumosilicates; they consist of linked SiO_4_ and AlO_4_ tetrahedra, thereby comprising a stable 3D-structure with open cages and channels [9,10]. Because of their porous structure and their negatively charged crystal framework, zeolites may bind different substances (including molecules) either on their surface (adsorption) or inside their porous structure (absorption), depending on the size and charge of the absorbed matter. Sometimes, both adsorption and absorption can occur concomitantly. Moreover, zeolites possess an ion-exchange capacity for certain metals and molecules. Zeolites are mined from natural deposits or synthesized with special features for distinct applications. They have been used for a long time and in various applications—for instance, as food/feed additives for humans or animals, as odor control agents in livestock farming, as soil fertilizers in agriculture and soil amendment, as filters for water, air, and waste, in technical applications such as chemical sieves, as building materials (such as cement), and in brick production [11,12].

As zeolites are known for their excellent sorption qualities, the question was raised whether they might also be able to bind gluten, which are especially harmful for people dealing with intestinal issues, such as celiac disease and gluten allergy.

In the experiments presented in this paper, purified clinoptilolite-tuff (PCT) was used. The mineral clinoptilolite, a form of natural zeolite classified as a heulandite-type, is the main mineral constituent of volcano-sedimentary tuff. The raw material of PCT, clinoptilolite-tuff, originates from a mine in the eastern Slovak Republic—a source that already contains lesser amounts of heavy metals than other clinoptilolite-tuff deposits. However, to make this high-grade raw material even safer, more effective, and well-tolerated for prolonged human consumption, it is purified according to a fully quality-controlled and patented process involving ion exchange, micronization, and terminal heating. The final product is marketed in the USA as G-PUR^®^ [9,10,13], and is herein simply termed as PCT (purified clinoptilolite-tuff). PCT is characterized in more detail in the Supplementary Table S1 and Figure S1.

Purified clinoptilolite-tuff can sorb different kinds of substances. In a clinical trial, it was described as a safe and highly effective binder for lead in drinking water [14] and as sorbent for health-relevant heavy metals in vitro [15]. Therefore, the binding of prolamins from food seems to be reasonable. Experiments were performed to determine whether, and in what amounts, gluten could be bound onto PCT. Removing gluten contaminations from gluten-free foods and providing people suffering from enteropathies with the opportunity to consume products containing traces of prolamins would enrich their diets and support the improvement of their clinical symptoms; mucosal recovery could be propagated and new damage could be avoided. Moreover, the social lives of such people could be ameliorated, as a less-restrictive diet could be followed and the threat of traces of potentially hazardous gluten would be less. Our investigations revealed a high sorption capacity of at least 20 mg gliadin to 1 g of micronized, purified clinoptilolite-tuff. In vitro data suggested that 2 g of PCT ingested daily could aid in inactivating up to more than 40 mg of gliadin (corresponding to 80 mg of gluten). With the increasing prevalence of prolamin-related disorders, a non-toxic, easily applicable, and well-tolerated dietary supplement could help patients to change their lives for the better.

## 2. Results

### 2.1. Adsorption of Gliadin on Purified Clinoptilolite-Tuff

First, experiments were performed to investigate whether gliadin binds to PCT and, if so, to define the conditions of optimal adhesion/adsorption.

#### 2.1.1. The Kinetics of Gliadin Adsorption to PCT

**Figure 1:**
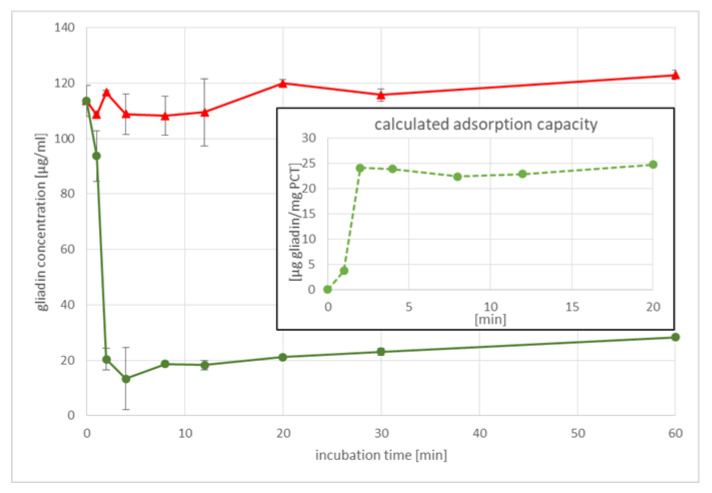
Incubation time versus gliadin concentration. A typical experiment is given. The red top line refers to the amount of gliadin in controls, while the bottom line in green shows the decline of gliadin in the supernatants in a time-dependent manner. Standard deviations are depicted for each time point and curve. In addition, the corresponding adsorption capacity is given by the dashed light green line (inserted diagram).

To analyze the binding characteristics in a time-dependent manner, gliadin and PCT mixtures were prepared. As first, the experiments revealed that almost all gliadin was adsorbed by purified clinoptilolite-tuff within 1 h of incubation at 37 °C, and the following tests were completed within much shorter times, beginning with the longest time point at 20 min and minimizing the incubation time down to 1 min for the last set of samples. As depicted in Figure 1, as few as 4 min were sufficient for binding the predominant amount of gliadin provided to PCT, as only 20 µg of gliadin were found in the supernatants by ELISA analysis.

Furthermore, the adsorption capacity was calculated. It was about 20–25 µg of gliadin per gram of PCT, which was typically found in this experimental setting.

#### 2.1.2. The Adsorption of Gliadin at Varying Particle Size Distributions

To test the potential of different particle sizes in adsorbing gliadin, three particular grain-size fractions of PCT were chosen (see Section 4.3). For in-depth analysis of the binding capacity of PCT (standard), two fractions (coarse and fine) of PCT were separated by centrifugation and used for the experiment. Moreover, another coarse grain-size fraction (defined as coarse powder) was also tested.

**Figure 2:**
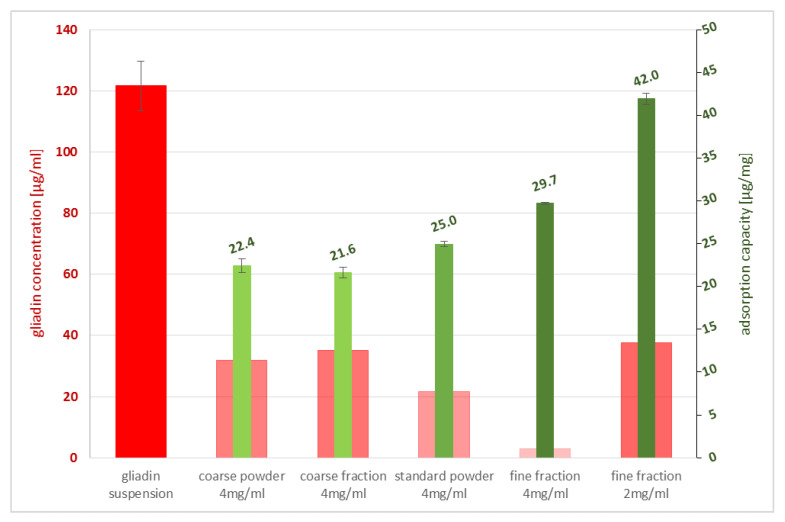
Gliadin concentrations in supernatants after incubation with different purified clinoptilolite-tuff fractions and adsorption capacity of different PCT fractions and concentrations concerning gliadin binding. PCT was used in different grindings to define the most sufficient size for gliadin binding by ELISA. The diagram illustrates the amount of gliadin found in the supernatants after incubation (wide red columns). Every single corresponding narrow green bar gives the specific binding potential of gliadin to purified clinoptilolite-tuff in [µg/mg].

As depicted in Figure 2, the fine fraction can bind most of the gliadin. While the wide columns show the amount of gliadin found in the supernatant after incubation of the various fractions with gliadin, the narrow columns show that 4 mg of the fine fraction have a binding capacity of 29.7 µg/mg gliadin on PCT, while 4 mg of the coarse fraction yields only 21.6 µg/mg.

The highest adsorption corresponds to 2 mg of the fine fraction, which is up to 42 µg/mg gliadin on PCT. Due to the relation of PCT weight to gliadin amount, the resulting value for 2 mg is higher than the value for 4 mg of the corresponding fraction.

The incubation time was 30 min.

#### 2.1.3. The Influence of pH on Gliadin to PCT Binding

Two different testing methods concerning miscellaneous pH values from slightly alkaline to highly acidic were used for the analysis of gluten adsorption on PCT. One method is described in the European Pharmacopeia [16], and the other one can be found in the EURL Evaluation Report on the Analytical Methods submitted in connection with the Application for Authorisation of a Feed Additive according to Regulation (EC) No 1831/2003 [16,17].

The influence of the pH value was determined by incubating PCT and gluten in different buffers and thereafter measuring the remaining unbound gluten by ELISA.

**Figure 3:**
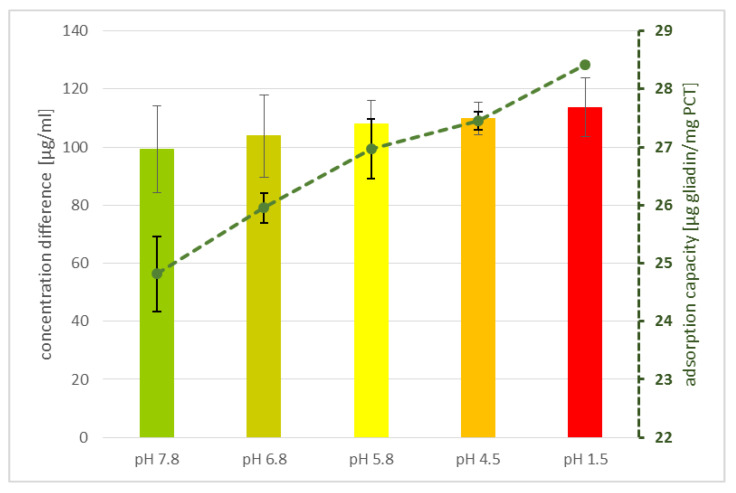
Adsorption of gliadin to PCT and corresponding adsorption capacity at varying pH. Different phosphate buffers (pH 4.5, 5.8, 6.8, and 7.8) and a test solution (pH 1.5) were used to determine the influence of pH in a binding experiment of gliadin (125 µL/mL) onto PCT (0.1 g/mL). One representative experiment is shown. Bars indicate the difference in concentration of gliadin measured in the pure gliadin solution and the amount of gliadin after incubation with PCT at each tested pH value corresponding to the “concentration difference” in the axis label. Every single bar gives the specific binding potential of gliadin to PCT in µg/mg. Standard deviations are shown. The dashed line relates to the calculated adsorption capacity.

Interestingly, the pH had only a minor influence on the detection of gluten, indicating that the adsorption and the binding capacity of PCT were only slightly influenced by the pH (Figure 3). Nevertheless, a tendency was seen—the higher the acidity of the solution, the lesser the free gliadin. The lowest amount of unbound gliadin was detected at pH 1.5 corresponding to 28.4 µg/mg adsorption capacity, compared to 24.8 µg/mg at pH 7.8.

To exclude any effect of the pH in the supernatant dilutions (1:100 in the sample dilution buffer), as is mandatory for ELISA procedure, the single dilutions were determined by pH measurement, revealing values in a range of pH 7.41–7.57. Thus, it was concluded that the sample dilution buffer had no impact on the outcome.

### 2.2. Adsorption of Gliadin on Purified Clinoptilolite-Tuff in Synthetic Gastric and Intestinal Fluids

**Figure 4:**
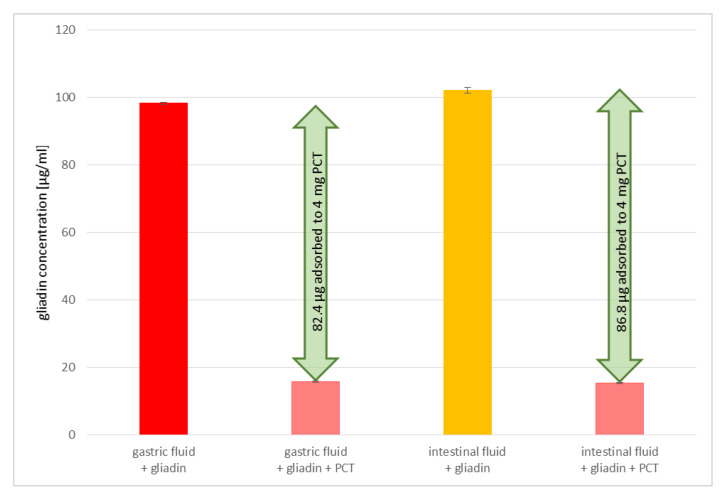
Gliadin bound to purified clinoptilolite-tuff in artificial fluids. Gliadin solutions were mixed with gastric or intestinal artificial fluids with and without PCT and analyzed by ELISA. Both approaches revealed adsorption capacities higher than 20 µg/mg gliadin to PCT.

Gliadin of a concentration of 100 µg/mL was incubated for 1 h at 37 °C rotating with and without 4 mg/mL PCT in synthetic gastric and intestinal fluids; both liquids were previously inactivated enzymatically by heat. The ELISA performed afterwards revealed an adsorption in both artificial liquids to an amount higher than 20 µg gliadin/mg PCT (20.6 µg/g for the synthetic gastric fluid and 21.7 µg/g for the synthetic intestinal fluid). As clearly shown in Figure 4, the adsorption of gliadin to PCT in artificial fluids was not hindered.

The thermal inactivation of pepsin and pancreatin was a crucial step in gaining results, as both enzymes undermine the ELISA procedure.

### 2.3. Adsorption of Prolamins Isolated of Barley and Rye on Purified Clinoptilolite-Tuff

The prolamins of barley (hordeins) and rye (secalins) were isolated from the respective flour/powder. As described for gliadins, the adsorption of secalins and hordeins to purified clinoptilolite-tuff was tested. A prolamin concentration of 50–150 µg/mL was used, and PCT was applied in two different fractions as fine and standard particle sizes in concentrations of 2–4 mg/mL.

**Figure 5:**
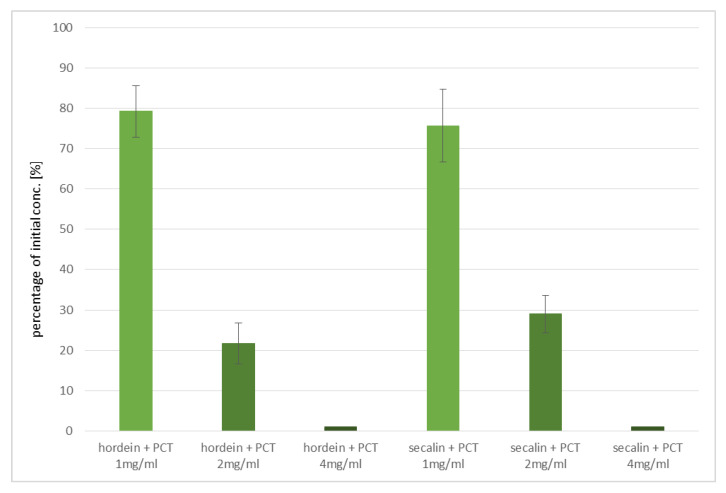
Prolamins from barley and rye adsorbed to purified clinoptilolite-tuff. Solutions containing either secalins or hordeins were incubated at 37 °C with and without PCT for 60 min. Then, the remaining prolamins in the single solutions were determined by ELISA.

It was found that more than 31 µg of hordeins and 47 µg of secalins bound to 1 mg of PCT, respectively (Figure 5). The adsorption capacity followed a specific kinetics, as the amount of bound prolamins increased characteristically for each type (secalins/hordeins/gliadins) when the dosage of PCT was increased. As shown in Figure 5, twice the amount of purified clinoptilolite-tuff absorbed nearly a doubled quantity of hordeins, while in the case of secalins it was only about 1.5×. This was verified in three independent experiments, each with quadruplicates. No adsorption capacity could be determined for the incubation of prolamins with 4 mg of PCT, as the detection limit of the ELISA was undercut.

All values multiplied by 2 reflect the adsorption capacity of gluten to purified clinoptilolite-tuff, as it is agreed that the ratio of prolamins and glutelins corresponds to 1:1 and gluten are seen as the sum of both. However, the ELISA method that was used only detected prolamins.

## 3. Discussion

Persons suffering from celiac disease, wheat allergy, and non-celiac gluten-sensitivity show very severe intestinal effects due to prolamins. Each milligram of gluten plays a decisive role for the well-being of such people. In particular, prolamins derived from wheat, barley, and rye cause the most severe problems. Other species of Poaceae (=Gramineae), however, may also contain gluten. Among them are spelt (*Triticum aestivum* subsp. *spelta*), Kamut (Khorasan wheat, *Triticum turgidum* × *polonicum*), the generic hybrids of wheat and rye called *Triticale* or *Triticumsecale* and *Secalotri(ti)cum*, as well as einkorn (*Triticum monococcum*) and emmer (*Tritium diococcum*), both of which are ancient grains that are very popular in modern diets [6,18]. The processing of all of these crops has a negligible impact on the hazardous effect of the gluten proteins. Avoiding those irritants by strictly following a gluten-free diet is of crucial relevance, as there is no cure for these diseases yet. However, traces of prolamins can be found even in products classified as gluten free, and, therefore, gluten cannot be completely avoided by affected individuals. Several steps, derived from agricultural techniques from harvesting to food processing, can be responsible for the contamination [6]. Thus, a dietary supplement binding the toxic plant peptides might be of great relevance for patients suffering from gluten proteins. This approach is particularly important because the gluten-free diet offers fewer vitamins and minerals, when compared to conventional food consumption. Therefore, slightly less-restrictive nutrition could offer great benefits concerning diet-related deficiency signs. Moreover, trapping triggers might help patients to recover and lead less painful lives.

For all experiments presented in this study, a special purified clinoptilolite-tuff (PCT), intensively tested and approved for prolonged human application [14,15,19], was used. Due to a patented purification process, PCT is free of toxic elements and safe for supplemental oral intake [13].

Clinoptilolite belongs to the family of natural zeolites, and has been used for decades for farm animals as well as for pets. In the European Union, clinoptilolite-tuff is allowed as feed additive [12,16,17,20]. In the United States of America, the raw material of PCT, clinoptilolite of sedimentary origin, is generally recognized as safe “for use as an anticaking agent in diets for cattle, swine, goats, sheep, poultry, cats and dogs at a level up to 1% by weight in complete diets” (GRAS Notice No. AGRN 29, 30 August 2019). Interestingly, the first specific tests on animals were performed in Japan in 1965, when natural zeolites were used as dietary supplements for ruminants, swine, and poultry. Subsequent scientific investigations showed a significant increase in feed efficiency and in the general health of livestock, with no apparent side effects [12,21,22]. Various studies applying low doses of zeolite-tuff (0.2–5% clinoptilolite-tuff/kg feed), combined with mycotoxin-contaminated feed, revealed remarkable results by preventing mycotoxin-related issues (mycotoxicoses; [22]). For an average person with 80 kg bodyweight, a daily application of 2 g PCT corresponds to 0.0025% PCT/kg, compared with an animal receiving 0.2–5% zeolite-tuff/kg at each feed. The in vitro experiments of this study showed a binding capacity of more than 20 mg gliadin per gram of PCT, which accords with a daily application of 2 g PCT for 40 mg gliadin, or an 80 mg gluten minimum-sorption capacity. Taking into consideration that less than 20 µg/g are defined as “gluten-free” (≙10 µg/g gliadin), 2 g of PCT can bind traces of prolamins sufficiently and could contribute to a safe diet.

The size of a particle’s surface is directly related to its adsorption capacity—the larger the surface, the higher the capability of sorption. Studying materials with varying particle-size distributions confirmed the highest gluten sorption capacity for the finest grain-size fraction. Hence, milling is a crucial step during the manufacturing of zeolite-based materials, as high-impact grinding tends to destroy the clinoptilolite’s crystal framework, and therefore lowers its efficacy. PCT is micronized with a special jetmill technique, resulting in a homogenous, narrow, and fine-grained range of particle-sizes with fully intact crystal structures; this allows the finished product to have the optimal binding characteristics. The lowest sorption capacity was obtained by the coarse fraction, which was used for the calculation above to demonstrate the impressive quality of gluten adsorption even under non-optimal particle sizes of PCT. Due to the effective pore size of PCT, corresponding to 0.4 nm [23], and our findings that the same weight of different fractions but divergent sizes of particles show incoherent sorption capacities, we strongly propose adsorption mechanisms, rather than absorption of prolamins onto PCT. This proposal is supported by the fact that the average particle size of gliadin is 3.88 nm, while that of hordein is 4.32 nm and that of secalin is 5.79 nm [24]. In addition, in-house XRD measurements before and after adsorption of gluten to PCT revealed no changes in the diffraction pattern. This was expected, as the relatively big prolamin proteins are not capable of either entering the pores or the crystal lattice.

Zeolites have been applied for decades in storage mold management. Bodroža-Solarov et al. [25] reported that the addition of zeolite to wheat dough through clinoptilolite-tuff containing residues in flour was problematic in changing the rheological properties of the wheat dough; they referred to a Russian patent that described the sensory properties of bread as deteriorating when exceeding 5% zeolite-tuff. Seen from this point of view, adding zeolite to prolamin-containing food seems to be inferior to oral administration. Moreover, the changing of the flow characteristics of the dough when zeolite is added reveals an action between the mineral and the gluten, as prolamins define the breadcrumb and are responsible for the texture and the elasticity of the dough. Casually speaking, gluten glues the ingredients of the dough together. This finding serves as a further indication of a specific binding of gluten to zeolite.

Another aspect of celiac disease is a deviation in the intestinal microflora, which is indicated by low diversity and aberrant composition [1]. When analyzing various manifestations of celiac disease in biopsy samples of patients showing intestinal and extraintestinal (anemia, dermatitis herpetiformis = Duhring’s disease) forms, the expression in the gastrointestinal tract or anemia was linked to lower diversity, moreso than when dermatitis herpetiformis was the main clinical symptom. Patients with such dermatological characteristic had similar intestinal microbiota as control subjects [26]. Another human study revealed that even treated celiac patients obeying a strict gluten-free diet showed persistent symptoms when their duodenial microbiota were altered. Proteobacteria were increased, and Bacterioidetes and Firmicutes were reduced, as their whole microbial richness was decreased [27]. Interestingly, Prasai et al. described the effects of natural zeolite-enriched feedstuff in poultry, when cloacal swabs indicated a significant reduction of proteobacteria without influencing the beneficial bacteria [28].

In this microbial context, bacteria—especially some of those isolated from curd and sourdough—are subjects of investigation regarding their ability to break down gluten by their metabolic activity. Scientific research on this topic started some decades ago, and deals with the definition of the special bacterial species responsible for this process, as high microbial diversity is found in both sourdough and curd [29]. *Bacillus* sp. [30], as well as species of Enterobacteriaceae and *Streptococcus* [31], were identified as showing proteolytic activity towards gluten proteins. These bacteria use gluten as carbon and nitrogen sources [31]. Interestingly, Enterobacteriaceae belong to the group of proteobacteria, while the genera *Streptococcus*, *Enterococcus*, and *Lactobacillus* are Firmicutes, which are described as advantageous for human health. The idea is either to administer gluten-hydrolyzing bacteria as probiotics (like *Bacillus* sp.) to reduce the gluten content of food directly in the human digestive tract or to add them to ferment the gluten-containing dough [30,31,32]. However, research is still at the pre-clinical level. 

Contrary to the intended use of bacteria in gluten degradation, the intake of PCT is well-established and easy to handle. Our results demonstrate the constant efficiency of gluten binding under different conditions (stomach/intestine); the mineral remains sorptive for gluten at various pH levels and artificial fluids, while bacteria have to survive the low pH of the stomach to reach their target location in the intestine, where the pH is in the alkaline range. In addition to changing pH levels, bile acid is another factor that bacteria have to overcome, as high levels of it are found in the small intestine. Furthermore, most bacteria are stored best under refrigeration for only a certain amount of time; PCT, however, can be kept at room temperature and in a dry environment with, theoretically, no expiration date. Using bacteria in sourdough for gluten degradation, a certain incubation time and temperature are necessary to gain an acceptable reduction of gluten, a process that must be considered in terms of profitability. PCT not only binds gluten irreversibly, as the studies on sorption kinetics revealed, but it is also extremely fast; within the very first 4 min, gluten adhered to zeolite-tuff. This reduces the risk of gluten-induced damage caused by a long retention time in the intestine.

A further approach to break down gluten is the use of enzymes—derived from either bacteria, fungi, insects, humans, or plants—in the gastro-duodenal tract before reaching the small intestine [33,34]. Although the main interest is on endopeptidases, there are some exopeptidases documented in the literature, as well [33]. Among the endopeptidases are glutenases, subtilisins, and propyl endopeptidases; synthetic glutenases are also created. Even human saliva is under investigation, as it contains a wide variety of bacteria, most of them descending from the phyla of Bacteroidetes and Actinobacteria [34]. To be useful for oral enzyme therapy, the potential candidates must be safe for human consumption and cleave prolin- and glutamine-rich domains; their structural stability and enzymatic activity over a broad pH range (highly acidic in the stomach and mildly alkaline in the intestine) must be maintained; and, most importantly, no increase of immunogenicity by producing small immunogenic peptides may occur [33,34]. Enzymes must work fast, as gluten should be degraded before they can cause harm in the small intestine. The utilization of several different enzymes combined as a drug could result in a synergistic effect, as some enzymes are already active at low pH in the stomach, whereas others show highest efficacy in the duodenum, which would increase the degradation time [33,34]. In some cases, coating with PEG and microencapsulation with PLGA helps to retain the enzymes in their active state. Research also focusses on molecular modeling through genetic modification to transform natural proteins into more stable, pH-resistant enzymes with higher substrate specificity and increased activity. However, as many factors must be taken into consideration, obtaining acceptable results from clinical studies has proved to be a difficult task [34]. An in vitro study by Tanner was published using nine commercially available digestive enzyme supplements to test their potential in the removal of celiac-relevant epitopes [35]. The test conditions included two pH levels, at 3.5 and 7.0. Gliadin was isolated and purified from a single wheat cultivar (cv. Baxter) and afterwards digested with trypsin and pepsin to gain immunoactive peptides, as takes place in human digestion. The results demonstrated unequal activity and reaction times of the single supplements at the pH values tested, as well as strong to no activity in rendering gliadin that were harmless. Only one, an oleoresin- (caricain) containing supplement, could efficiently and rapidly degrade the trypsin/pepsin pre-digested gliadin at pH 7 [35]. Sometimes, a mixture of enzymes must be applied, because only in combination can they offer their full, non-human-toxic action as described for *Pseudomonas*/*Lactobacillus* (while the first one cleaves gluten, resulting in smaller immunogenic peptides, the second one fragmentizes them into non-immunogenic gluten-residues) [34] or *Aspergillus niger*/*Aspergillus oryzae* (aspergillo-pepsin and dipeptidyl peptidase IV cooperatively degrade gluten) [33,34]. Taking all noted factors into account, enzymes can provide help in the future for gluten-sensitive persons. Regarding the unrestricted gluten-binding capacity throughout the various pH levels in the whole intestinal tract, as tested in this work (starting at pH 1.5 and ending at pH 7.8), the PCT bears some significant advantages toward enzymes. Furthermore, the straightforward administration, without any indispensable coating, also contributes to PCT being favored over enzymes.

In addition, the adsorption of gluten to PCT seems to be relatively specific, as demonstrated in experiments with artificial fluids. Although there is a higher concentration of denaturated proteins (trypsin and pepsin, among others) than gluten in the solution, the binding of prolamins to PCT is not hindered, as revealed by ELISA. Perhaps a partial charge of the prolamins and/or their poor solubility in aqueous solution are responsible for the effect observed. Previous findings endorsed this presumption, as shown, e.g., for *Clostridioides difficile* toxins A and B, which were bound to PCT and thus neutralized [19]. Previously, it was known that these toxins have an affinity with an anionic resin called tolevamer, a polymer used for the treatment of *C. difficile*-associated diarrhea in humans [36]. Zeolites are composed of SiO_4_ tetrahedra with an isomorphic substitution of aluminum ions, thereby inducing a negative framework charge and generating a cation-binding capacity [37].

In the experiments performed, purified zeolite-tuff effectively sorbed the most celiac disease-critical prolamins of wheat (gliadins), barley (hordeins), and rye (secalins). As bread is often made of mixtures of wheat and rye, this result is a key advantage. Binding of barley’s hordeins is another asset of purified clinoptilolite-tuff, because pearl barley is a popular food. Since cross contaminations of the three different grains can occur easily during processing, a universal and non-specific prolamin-adsorbing capacity is a decisive positive factor in favor of PCT, especially since there are no negative side-effects documented, even when taken over longer periods.

Concerning the distinctive adsorption of the single prolamins, this effect might be based on their unique characteristics. Rani et al. vigorously studied the particular forms of prolamins of wheat, barley, and rye. They found, by applying scanning as well as transmission electron microscopy, that both the surface morphology and the shape of single gluten proteins differed specifically [24]. Further, Lexhaller et al. compared prolamins and glutelins derived from wheat (cv. Akteur), barley (cv. Marthe), and rye (cv. Visello) with five sandwich ELISA kits [38]. Thus, in addition to the different structures of gliadins, hordeins, and secalins [24], inconsistent gluten values might be due to different affinities of the R5 monoclonal antibody of the ELISA kit used for detection, as shown by studies using various ELISA kits and the RP-HPLC technique. A higher sensitivity of the R5 monoclonal antibody toward secalins and hordeins, when compared to the sensitivity to gliadin, was demonstrated [38]. 

A collaborative study concerning the “determination of gliadin as a measure of gluten in food by R5 Sandwich ELISA RIDASCREEN gliadin matrix extension” included 14 participating laboratories. The recovery rate was 80–130% and the relative reproducibility standard deviations for the contaminated samples were between 9.8% and 27.7% [39]. In a review, Shewry noted that even wheat cultivars might give different gliadin results, as the number of epitopes for ELISA antibodies can vary among the single varieties when summarized in comparative studies of ancient and modern wheats. In fact, wheat samples grown under different conditions might exhibit other characteristics, and thus adverse effects for sensitive persons [40]. Taking all these findings together, our results show the obviously prolamin-binding of PCT. Whether or not the higher adsorption values are attributable to the higher affinity of secalins and hordeins onto PCT, or are based on more binding sites (epitopes) than found in gliadin, is not deducible.

Taking all these considerations into account, our in vitro data clearly suggest that the intake of PCT could be highly beneficial for persons suffering from gluten-induced diseases. However, we are aware that only a clinical trial can thoroughly elaborate the positive effects of PCT administration on gluten-linked disorders.

## 4. Materials and Methods

### 4.1. The Chemical Composition of Buffers Used in the Experiments Performed

Buffers of different kinds were used for various experiments. In Table 2, an overview of the specific buffers, including their single components, concentrations, and pH values, is provided (according to European Pharmacopoeia 10.0, Chapter 2.9.3, [16]).

### 4.2. Synthetic Fluids Preparations

Both artificial fluids were prepared according to the recommendations of the European Pharmacopoeia [16] and as noted in the EURL Evaluation Report [17] on the Analytical Methods submitted in connection with the Application for Authorisation of a Feed Additive according to Regulation (EC) No 1831/2003 [16,17].

#### 4.2.1. Preparation of Artificial Gastric Fluid

Pepsin (P7000, Sigma, Kawasaki, Japan) in the amount of 3.2 g was dissolved in 500 mL of distilled water prior to inactivation at 95 °C for 1 h. This was crucial in performing ELISA testing, as previous experiments revealed that pepsin altered the structures of prolamins so that they were no longer captured by the antibodies provided in the ELISA kit. Afterwards, 2.0 g NaCl (sodium chloride, 71380, Sigma-Aldrich, St. Louis, MO, USA) were added and dissolved before 80 mL of HCl [1 M] (hydrochloric acid 25%, CVH Chemie-Vertrieb GmbH & Co, Hannover, Germany) were pipetted to the solution. Finally, distilled water was added to reach a final volume of 1000 mL. The measured pH value was 1.3 at room temperature (pH 3210i, WTW).

#### 4.2.2. Preparation of Artificial Intestinal Fluid

Pancreatin (P1750, Sigma, Kawasaki, Japan) in the amount of 10 g was dissolved in 500 mL distilled water before inactivation at 95 °C for 1 h. As described for the preparation of synthetic gastric fluid, this step was important for analyzing samples by ELISA. Next, 250 mL KH_2_PO_4_ [0.2 M] were mixed with 77 mL NaOH [0.2 M] (sodium hydroxide solution, ≥32%, extra pure, Carl Roth, Karlsruhe, Germany) and added to the dissolved pancreatin solution. Then, the pH was fixed to 6.8 and distilled water was used to reach an end volume of 1000 mL.

### 4.3. Measurement of Particle Size Distribution

**Figure 6:**
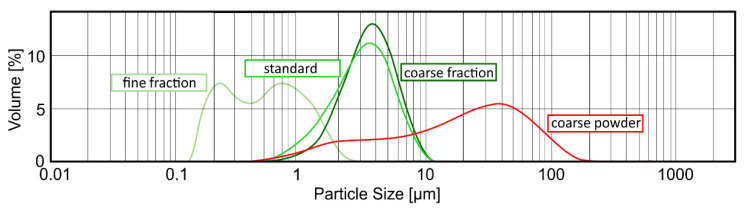
Different particle size distributions of PCT used for gliadin binding. The distribution of various particle sizes was analyzed by Mastersizer 2000 measurement and evaluated with software version 5.31 (Malvern Instruments).

The determination of the sorption capacity is a crucial step in analyzing the characteristics of purified clinoptilolite-tuff. Generally, the smaller the particle is, the larger the surface, and hence the sorption capacity, are (valid for molecules bigger in size than the pores of the PCT). To study this phenomenon in detail, three different grain-size fractions of G-PUR^®^ were examined. First, G-PUR^®^ was tested as a whole (standard), containing all particle sizes (d50 = 3.18 µm); furthermore, fine (d50 = 0.52 µm) and coarse (d50 = 3.57 µm) fractions of G-PUR^®^, which were separated by centrifugation from the initial product, were analyzed. The coarse powder (d50 = 20.90 µm) corresponding to the purified material of which G-PUR^®^ was produced, but this was carried out before micronization. The distribution of the examined single PCT samples is listed in Table 3 and illustrated in Figure 6.

The measurement of the particle size distribution using the Malvern instruments device Mastersizer 2000 was performed as follows. The sample was added till the light attenuation in the analyzer reached 10–20%. After 10 s of ultra-sonication and a 1 min incubation time, the sample was measured in triplicate and averaged. Three consecutive samples of each particle fraction were analyzed (i.e., nine measurements per PCT fraction). Data processing and evaluation was carried out via the Mastersizer 2000 software, version 5.31.

The procedure followed a quality-controlled, standardized, and validated method for routine analysis of PCT particle fractions.

### 4.4. The Kinetics of Gliadin Adsorption to PCT

To analyze the binding of gliadin to PCT in a time-dependent manner, an ELISA assay (RIDASCREEN^®^ Gliadin ELISA kit, R7001, R-Biopharm AG, Darmstadt, Germany) was used. Gliadin (gliadin from wheat, G3375, Sigma-Aldrich) was diluted in phosphate buffer pH 6.8 containing 250 mL of 0.2 M KH_2_PO_4_ (potassium dihydrogen phosphate, 26,931.263, VWR) and 112 mL of 0.2 M NaOH (sodium hydroxide solution, ≥32%, extra pure, Carl Roth) in a final volume of 1000 mL to a final concentration of 125 µg/mL. Purified clinoptilolite-tuff was diluted in the same buffer to gain an end-concentration of 4 mg/mL. Controls containing only gliadin in suspension were performed in duplets, while gliadin and PCT mixtures were set up in quadruplets. All samples were incubated at 37 °C under rotation (25 rpm/min, rotator SB3, Stuart) between 1 h as maximum and 1 min as minimum. The samples were centrifuged (MEGA STAR 1.6R, VWR) at 4100× *g* for 2 min at room temperature prior to determining the content of gliadin in the supernatants by ELISA, following the manufacturer’s instructions. Measurements were performed at a wavelength of λ = 450 nm by the use of a plate reader (Biotek synergy HT, BioTek Instruments, Inc., Winooski, VT, USA).

### 4.5. Adsorption of Gliadin to Different PCT Fractions

The prolamin solution containing gliadin from wheat (G3375, Sigma) was extracted according to the ELISA’s manufacturer’s advice, using the patented cocktail developed by Dr. Enrique Mendez (R7006, R-Biopharm AG, Darmstadt, Germany), which is the official method according to the AOAC. Then, the extract was diluted to a final concentration of 150 µg/mL in phosphate buffer pH 6.8 containing 250 mL of 0.2 M KH_2_PO_4_ and 112 mL of 0.2 M NaOH in a final volume of 1000 mL.

The different fractions (coarse, standard = G-PUR^®^, and fine) and a coarse powder of PCT were used in various amounts, as noted in Table 3. They were also diluted in phosphate buffer pH 6.8.

Prior to mixing, the various solutions were pre-heated to 37 °C to simulate human body temperature.

After 30 min of incubation rotating (25 rpm/min, rotator SB3, Stuart) at 37 °C, the samples were centrifuged at 4100× *g* (MEGA STAR 1.6R, VWR) for 2 min and the supernatants were analyzed by ELISA (R7001, R-Biopharm AG, Darmstadt, Germany), as recommended by the manufacturer, to examine their adsorption value. Measurements were performed at a wavelength of λ = 450 nm by using a plate reader (Biotek synergy HT).

### 4.6. The Influence of pH

The determination of a possible influence of acidity or alkalinity on gliadin adsorption to purified clinoptilolite-tuff was performed by incubation of 125 µg/mL gliadin and 0.1 g/mL PCT for 30 min at 37 °C under rotation (25 rpm/min, rotator SB3, Stuart) in different buffers, including pH 7.8, 6.8, 5.8, 4.5, and a solution of pH 1.5 (Table 2). Then, the samples were centrifuged at 4100× *g* (MEGA STAR 1.6R, VWR) for 2 min and the supernatants were analyzed by ELISA (R7001, R-Biopharm AG, Darmstadt, Germany), according to the manufacturer’s guidelines, to examine their adsorption value. Measurements were performed at a wavelength of λ = 450 nm by using a plate reader (Biotek synergy HT).

### 4.7. Determination of Gluten Content via ELISA

Gluten were detected by using the RIDASCREEN^®^ Gliadin ELISA kit (R7001, R-Biopharm AG, Darmstadt, Germany), which is especially designed for the quantitative analysis of wheat, rye, and barley prolamins in food declared as gluten-free. It is certified as the Codex Alimentarius Method (Type I) and by the AOAC. It has a limit of detection according to 0.5 mg/kg gliadin (≙1.0 mg/kg gluten) and a limit of quantification referring to 2.5 mg/kg gliadin (≙5.0 mg/kg gluten). Prolamins react with the monoclonal R5 antibody offered in the kit.

The gliadin content of the supernatants was determined using the RIDASCREEN^®^ Gliadin ELISA kit (R7001, R-Biopharm AG, Darmstadt, Germany) before measurement of the samples in a spectrophotometer (Biotek synergy HT) at a wavelength of λ = 450 nm.

It is important to note that the content of gluten refers to twice of the amount determined for gliadin/hordein/secalin. This is based on the assumption that the ratio of prolamin:glutelin is 1:1.

### 4.8. The Isolation of Secalins and Hordeins of Flour

Flour of barley and rye was used for the isolation of hordeins and secalins, respectively.

Organic barley and organic rye were purchased from different providers, either as grain or as flour. Organic barley (Rosenfellner Mühle, St. Peter in der Au, Austria, MHD 080720219 and Spar Österreich Warenhandels-AG, Salzburg, Austria, L22013S0421, MHD 31.01.2023) and organic rye (Rosenfellner Mühle, MHD 02042021) grain were rough-ground in a kitchen appliance (Russel Hobbs, Multifunctional Blender) before milling in a vibrational ball mill (MM 400, Retsch, Hahn, Germany) at a frequency of 30 Hz for 1 min. The resulting powder of the milled barley and rye was sieved (250 µm sieve) in a vibratory sieve shaker (AS 200 control, Retsch) and the fine fraction (<250 µm) was used for the extraction. The single powders or the purchased rye flour (Spar Österreich Warenhandels-AG, Typ 960, L215087, MHD 17.10.2022) were then incubated with the patented cocktail (R7006, R-Biopharm AG, Darmstadt, Germany) according to the manufacturer’s guidelines to isolate the specific prolamins. In short, 500 mg of the barley or rye samples were extracted by the addition of 5 mL of the patented cocktail at 50 °C in a water bath (WB 10, Medingen, Germany) for 40 min. Afterwards, a cooldown of the samples at room temperature lasted for 3 min. Then, 15 mL of 80% EtOH were added per sample and mixed thoroughly (vortex mixer SA8, Stuart). Another incubation rotating (25 rpm/min, rotator SB3, Stuart) at room temperature lasted for 1 h. Finally, the samples were centrifuged two times, first at 3000× *g* (MEGA STAR 1.6R, VWR) for 10 min and subsequently at 21,885× *g* (Biofuge primo R, Heraeus, Hanau, Germany) for another 10 min. The resulting supernatants containing the extracts were transferred to a new vial and stored at room temperature in the dark.

The concentration of prolamins in the resulting extracts was determined by RIDASCREEN^®^ Gliadin ELISA (R7001, R-Biopharm AG, Darmstadt, Germany).

Binding experiments of secalins and hordeins to purified clinoptilolite-tuff were performed, as described for gliadins.

## 5. Conclusions

An in vitro study was performed to elucidate the binding capacity of gluten onto purified clinoptilolite-tuff, a special type of zeolite that was purified by a patented technique after mining, being micronized, and being terminally heated.

All incubations of single experiments were conducted at 37 °C to mimic human body temperature. To imitate the peristalsis, and hence the movement of the PCT suspension in the intestine, a homogenous PCT suspension, either in water or in artificial fluids, was maintained during the experiments by vortexing, allowing no gravitational settling of the particles. The pH levels of the human gastrointestinal tract were relevant to the exploration. It was shown that the binding of prolamin to PCT was not significantly affected at pH 1.5 to 7.8, indicating that neither the acidic milieu of the stomach nor the alkaline of the intestinal tract hinders the sorption of gluten to PCT. This was further confirmed by the use of both artificial gastric and intestinal fluids. Both synthetic fluids containing more denaturated proteins than gluten displayed the specific reaction of prolamins and PCT, as no impediment of adsorption could be found by ELISA analysis. Moreover, studies of the sorption kinetics confirmed that bound gluten irreversibly adhered to the PCT, as no release was detected in the time course tested.

The adsorption to PCT took place within a very short time—as kinetics revealed, 4 min were sufficient to clarify all gliadin of the test solution. Interestingly, different adsorption capacities were found for each of the single prolamins of the wheat, barley, and rye tested. Following specific kinetics of each prolamin analyzed, binding of up to 25 µg/mg gliadin (≙50 µg/mg gluten) of wheat were verified, while under the same conditions, 31 µg hordeins (barley) and 47 µg secalins (rye) could be adsorbed to 1 mg of PCT.

## Figures and Tables

**Table 1:** Gluten consists of a mixture of proteins combining prolamins and glutelins. Their respective names are given to different crops [1,4,5]. While wheat, barley, and rye are known as gluten-containing crops that are toxic for patients with celiac disease, corn, rice, and sorghum are well-tolerated. Oat stands in-between, as it can induce severe problems or have no effect at all, depending on the individual person.

Crop (*Genus*)	Prolamin	Glutelin
wheat (*Triticum aestivum*)	gliadin	glutenin
barley (*Hordeum vulgare*)	hordein	hordenin
rye (*Secale cereale*)	secalin	secalinin
oat (*Avena sativa*)	avenin	avelanin
corn (*Zea mays*)	zein	zeanin
rice (*Oryza sativa*)	oryzin	oryzinin
sorghum (*Sorghum bicolor*)	kafirin	-

**Table 2:** Preparation of phosphate buffers and test solution. The single buffers were prepared from chemicals with the highest purification grade. Distilled water was used as solvent. pH values were fixed by the addition of either 0.1 M NaOH solution or 0.1 M HCl solution. The pH measurement was performed by the use of an electrode-pH meter with a temperature sensor (pH 3210i, WTW) at room temperature.

Phosphate Buffer [pH]	KH_2_PO_4_ Solution [0.2 M] Volume [mL]	NaOH Solution [0.2 M] Volume [mL]	Final Volume [mL]
7.8	250	222.5	1000
6.8	250	112	1000
5.8	250	18	1000
**Phosphate Buffer** **[pH]**	**KH_2_PO_4_** **[g]**		**Final** **Volume** **[mL]**
4.5	13.61		1000
**Test Solution** **[pH]**	**NaCl Solution** **[0.2 M]** **Volume [mL]**	**HCl** **[0.2 M]** **Volume [mL]**	**Final** **Volume** **[mL]**
1.5	250	207	1000

**Table 3:** Particle size distributions used to determine the different adsorption capacities of gliadin on PCT and the amounts used in experiments. The various fractions of G-PUR^®^ were determined as coarse, standard, and fine. Moreover, another coarser fraction was used, called coarse powder. Note that standard is the particle size usually used for experiments. Analyses were performed using a Mastersizer 2000 equipped with software version 5.31 (Malvern Instruments Ltd., Malvern, UK).

Type of PCT	Mean Value Particle Size [µm]			Amount Used in an Experiment [mg/mL]
	*d10*	*d50*	*d90*	
fine fraction of G-PUR^®^	0.20	0.52	1.30	0.25, 0.5, 1, 2, 4
standard = G-PUR^®^	1.36	3.18	5.95	4
coarse fraction of G-PUR^®^	1.85	3.57	6.26	4
coarse powder	2.28	20.90	70.20	4

## Data Availability

All data generated and analyzed are included in the published article.

## Supplementary Materials

The following supporting information can be downloaded at: https://www.mdpi.com/article/10.3390/ijms23095143/s1. Refs. [23,41] are listed in Table S1.
