# Supplementary material for: Purified Clinoptilolite-Tuff as an Efficient Sorbent for Gluten Derived from Food
Source: Int J Mol Sci. 2022 May 5;23(9):5143. doi: 10.3390/ijms23095143 (PMC9101028; doi:10.3390/ijms23095143)
Supplement: Supplementary file 1 [file ijms-23-05143-s001.zip › ijms-1706648-supplementary.pdf]

International Journal of *Molecular Sciences*

MDPI

Supplementary Material

Purified clinoptilolite-tuff as an efficient sorbent for gluten derived from food

Carmen Ranftler, Andreas Röhrich, Andreas Sparer, Cornelius Tschegg and Dietmar Nagl

6. Supplementary Material

6.1. The Chemical Composition and Physico-Technological Parameters of PCT

Table S1. Summarized chemical composition and physico-technological parameters of PCT

Composition and technological parameters of PCT				References
Loss on drying [%]	0.5	to	6	inhouse data
Loss on ignition [%]	3	to	8	inhouse data
Clinoptilolite content [wt%]	75	to	85	inhouse data
Ion exchange capacity [mol/kg]	0.75	to	1.10	inhouse data
Particle size d50 [µm]	3	to	3.5	inhouse data
Porosity [%]	24	to	32	[23]
Effective pore diameter [nm]		0.4		[23]
Surface area SBET < 0.125 mm [m^2g^{-1}]		59.2		[41]
Total dissolved solids [%]		< 0.15		inhouse data
pH-value	8	to	10	inhouse data
Al_2O_3 [%]	9.5	to	14	inhouse data
CaO [%]	2.6	to	6	inhouse data
K_2O [%]	0.4	to	1.8	inhouse data
MgO [%]	0.2	to	0.4	inhouse data
Na_2O [%]	0.2	to	1.3	inhouse data
SiO_2 [%]	65	to	75	inhouse data
As [µg/g]	0.5	to	1.5	inhouse data
Ba [µg/g]	40	to	70	inhouse data
Cd [µg/g]		< 1		inhouse data
Mn [µg/g]	5	to	25	inhouse data
Pb [µg/g]	1	to	2	inhouse data
Bioburden [CFU/g]		< 100		inhouse data
E. coli in 10 g		negative		inhouse data

Citation: Ranftler, C.; Röhrich, A.; Sparer, A.; Tschegg, C.; Nagl, D. Purified clinoptilolite-tuff as an efficient sorbent for gluten derived from food. *Int. J. Mol. Sci.* **2022**, *23*, 5143. https://doi.org/10.3390/ijms23095143

Received: 15 April 2022
Accepted: 2 May 2022
Published: 5 May 2022

Publisher's Note: MDPI stays neutral with regard to jurisdictional claims in published maps and institutional affiliations.

Copyright: © 2022 by the authors. Licensee MDPI, Basel, Switzerland. This article is an open access article distributed under the terms and conditions of the Creative Commons Attribution (CC BY) license (https://creativecommons.org/licenses/by/4.0/).

6.2. Scanning Electron Microscopy (SEM) Pictures of PCT

Secondary electron detector SEM pictures were taken of 2 different PCT fractions, standard (corresponding to G-PUR®) and the fine fraction of G-PUR®, to visualize the various particle sizes (Figure S1). The particle size distributions are documented in Table 2 and Figure 6 of the main text.

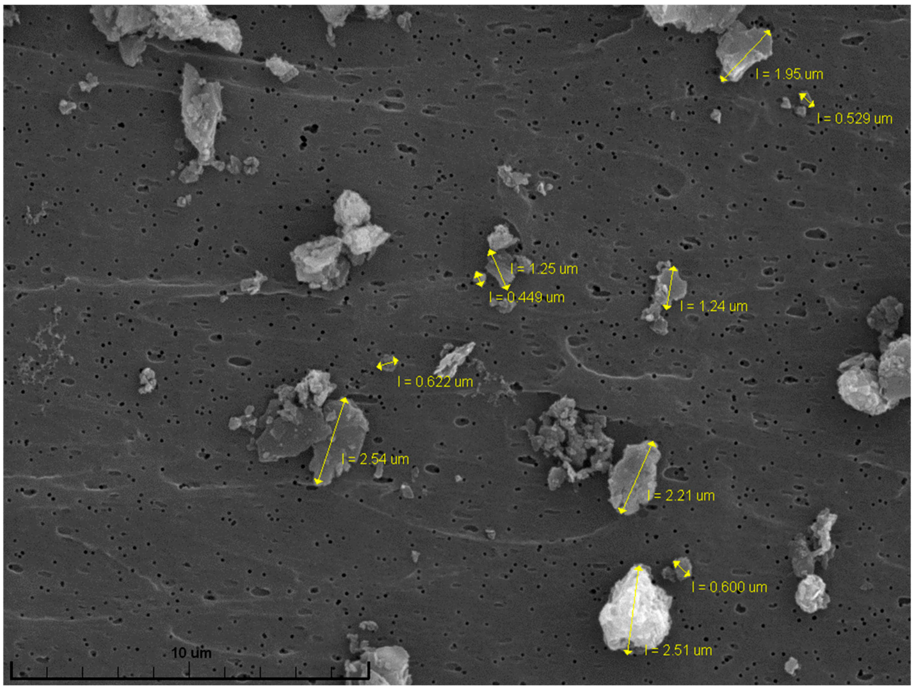

(**a**)

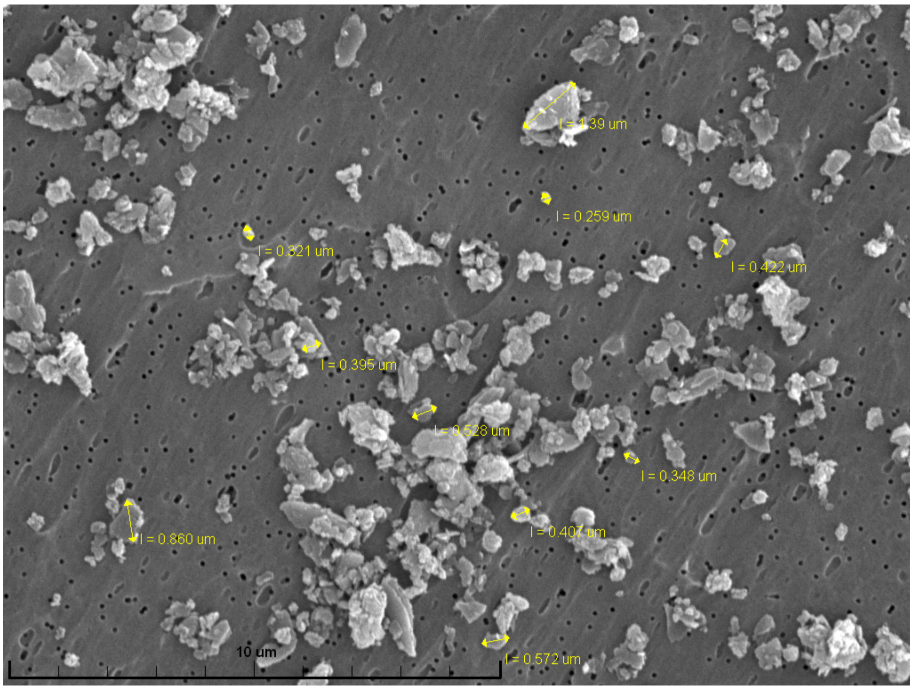

(**b**)

Figure S1. Scanning electron microscopy (SEM) pictures of PCT. (**a**) Standard fraction; (**b**) fine fraction of PCT. Both illustrations contain scale and length-measurements of single particles. The pictures were taken with a Vega Tescan scanning electron microscope, settings: 15.0 kV high tension, magnification 11.30 kx for standard fraction and 15.53 kx for fine fraction.
